# Risk factors of fatal occupational accidents in Iran

**DOI:** 10.1186/s40557-018-0241-0

**Published:** 2018-05-02

**Authors:** Hadi Asady, Mehdi Yaseri, Mostafa Hosseini, Morvarid Zarif-Yeganeh, Mahmoud Yousefifard, Mahin Haghshenas, Parisa Hajizadeh-Moghadam

**Affiliations:** 1 Social Determinants of Health Research Center, Saveh University of Medical Sciences, Saveh, Iran; 2 Department of Occupational Health Engineering, School of Public Health, Tehran University of Medical Sciences, Tehran, Iran; 3 Department of Epidemiology and Biostatistics, School of Public Health, Tehran University of Medical Sciences, Tehran, Iran; 4 PharmD-MPH, Faculty of Pharmacy, Tehran University of Medical Sciences, Tehran, Iran; 5 Department of Physiology, School of Medicine, Tehran University of Medical Sciences, Tehran, Iran; 6 Master of Occupational Health Engineering, Environmental & Occupational Health Center, Ministry of Health and Medical Education, Tehran, Iran; 7 Bachelor of Occupational Health Engineering, Environmental & Occupational Health Center, Ministry of Health and Medical Education, Tehran, Iran

**Keywords:** Fatal occupational accidents, Years of life lost, Iran

## Abstract

**Background:**

Occupational accidents are of most important consequences of globalization in developing countries. Therefore, investigating the causes of occupational accidents for improving the job situation and making operational policy is necessary. So the aim of this study was to investigate factors affecting the fatal occupational accidents and also calculate the years of life lost for dead workers.

**Methods:**

This cross-sectional study was conducted on data related to the 6052 injured workers that was registered in the 2013 registry system of the Ministry of Health and Medical Education of Iran. Variables including sex, education, age, job tenure, injury cause, referred location of injured workers, occupation, shift work, season, accident day, damaged part of the body were chosen as independent variables. The Chi-squared and Fisher exact tests were used for univariate analysis and then exact multiple logistic regression was carried out to identify independent risk factors of fatal occupational accidents. Finally, for dead workers, years of life lost, according to the injury causes was calculated.

**Results:**

Among the 6052 accidents reported, 33 deaths were recorded. Chi-square and Fisher exact tests showed that factors including: current job tenure (*p* = 0.01), damaged parts of the body (*p* < 0.001) and injury cause (*p* < 0.001) are associated with the fatal accidents. Also exact multiple logistic regression analysis showed a significant association between electric shocks as a cause of injury (OR = 7.04; 95% CI: 1.01–43.74; *p* = 0.02) and current job tenure more than 1 year (OR = 0.21; 95% CI: 0.05–0.70; *p* = 0.005) with the fatal accidents. The total amount of years of life lost based on causes of injuries was estimated 1289.12 years.

**Conclusions:**

In Iran, fatal accident odds in workers with job tenure more than 1 year was less in comparing to the workers with job tenure less and equal to 1 year. Also odd of death for electrical shock was more than other causes of injuries. So it seems that employing of workers who have more than one-year work experience in a specific job and using of appropriate safeguards will be useful for the reducing of fatal occupational accidents.

## Background

One of the most important consequences of globalization especially in the developing countries is occupational accidents, therefore, occupational health and safety issues remain a significant public health problem [1, 2]. Although major and important movements have been performed in protecting workers from occupational injury and illness, recent development has not been enough and much remains to be done. According to the findings of the ILO, every 15 s, a worker dies from a work-related accident or disease and also every 15 s, 153 workers have a work-related accident. The human cost of this daily adversity is vast and the economic burden of poor occupational safety and health practices is estimated at 4% of global Gross Domestic Product (GDP) each year [3]. Besides, many studies show that the total consequences of occupational injury and deaths is not just direct physical injury and these outcomes through lost productivity and increased use of medical and welfare services have a broad array of social and economic burdens [2, 4, 5].

Given the importance of the occupational accidents, statistics related to these accidents are published in many countries annually, however because of under-reporting, these data are not so authentic but provides useful insights [1, 6]. Also, analyses of these data with regard to socio-demographic factors, occupation types and industrial sectors have been especially useful in determining injury patterns and making operational policy for improving safety and health condition [2]. A study conducted by Smith et al. used data from the National Health Interview Survey indicated that injuries at work covers an significant portion of the total injury burden in the United States and in some age groups this portion was almost 50% of all injuries experienced [7]. Also, Gonzalez-Delgado et al. study showed factors including sex (being male), age, employed in the position for 1 to 10 years (versus less than 1 year), working as a facilities or machine operator or assembler and being a worker without qualifications (versus an office worker) were associated with fatal occupational accidents [8]. Barlas and Izci analyzed data related to shipyard occupational accidents that was registered in Ministry of Labour and Social Security of Turkey. They found that five major reasons for the fatal occupational accidents in this job are falling to a lower level, electric shock, fire and/or explosion, struck with and caught in between of objects, and drowning [9]. In this regard parameters like male gender, secondary educational level, causal employee, unskilled performance, and daytime duty work have been observed by Khodabandeh et al. as risk factors for fatality in the event of construction fatal injury [10].

Up to now there is no study neither to identify factors affecting the fatal consequence of occupational accidents using data from a national registration system nor estimated the Years of Life Lost (YLL) [11] of dead workers carried out in Iran. The YLL is a valuable and simple measure for estimating of economic burden of morbidity and mortality [12].

## Methods

### Design and setting of the study

This analytical cross-sectional study utilized data from the 2013 registry system of the Ministry of Health and Medical Education of Iran. This patented system of worker’s accident information was launched in 2013 for the first time in Iran with the help of medical universities and related health care units. The target population was industrialized and non-industrialized adults, aged 18 years or older. It worth mentioning that as their main outcome of interest was death but there was not a nationwide study before it. So, in order to increase the precision of estimates the proportion of death was preliminary assumed as 50% (*p* = 0.50) and with confidence level of 95% (α =0.05) for an error rate of 1.5% (d = 0.015) using $n=\frac{Z_{1-\alpha /2}^2\times p\times \left(1-p\right)}{d^2}$ the minimum sample size for this study was computed at least as 4269. Fortunately, they could gather data on 6052 individual accidents all over the country.

### Measures

The dependent variable in this study was the consequence of the accidents in injured people that were categorized into two groups including the survived and dead. The independent variables were including sex, age (a quantitative variable), education, tenure in current job (a quantitative variable), occupation, usual work schedule, injury cause, damaged part of the body, date of the accident and referred location of the injured people. While sex has two groups, male and female, for analyses, we grouped age into two groups: < 45 and 45 or older years and education into “Not graduate from high school” and “Graduated from high school”. Job tenure was assessed in terms of the number of years that injured people had held their current job. To investigation the relationship of this measure with depend variable it categories into two groups: 1 year or less and more than 1 year. About injured workers occupations (130 different occupations) variable nine different categories based on the 2010 U.S. Census Occupation Code were made [13]. Categories including day shift, evening shift and night shift were used for shift work variable. Injuries causes measure in the registry system of accidents data categorized as: falling, collision & throw & hit, trapping, excessive force, high or low temperature, electrical shock, acute cause and other causes but for analyzing goals this measure recoded into three groups including: falling & collision & throw & hit, electrical shock and trapping & excessive force & other reasons finally.

For damaged parts of the body variable seven groups including: upper extremity, lower extremity, trunk & wall, head & neck, abdomen & pelvis, thorax and other parts were used in the statistical analysis. Season of accident has four groups including spring, summer, fall and winter while for accident day variable three groups including 1–10, 11–20 and 21–31 were made. For referred location of the injured people in the recording system of accidents data six category including: work place healthcare center, urban or rural healthcare center, hospital, clinic, forensics and others were existed but for statistical analyses this measure categorized into the healthcare center, hospital and other groups.

### Data analysis

Data were analyzed by the Stata 14.0 (Stata Corp. USA) and R (version 3.3.1) software. Two sets of analyses were performed. First, univariate analyses were performed to assess the relationship of each potential risk factors with the consequence of occupational injury. For this purpose, Chi-Squared test, and Fisher exact test were used. Subsequently, an exact multiple logistic regression analysis was performed using the variables that were significant at *p* < 0.10 in the univariate analyses in order not to lose important variables due to confounding effect. As in this analysis, cells with small count was under investigation, exact procedure with backward elimination method was employed. In exact multiple logistic regression analysis first group of each variable was chosen as a reference group.

To calculate the YLL for each dead work force, age of death was subtracted from his/her life expectancy that it was derived from the life table of Iranian population [14]. One-way ANOVA test was used to compare mean of YLL related to each COIs (Causes of Injuries). Statistically significant differences were accepted at the *p* < 0.05.

## Results

Of the 6052 injured subjects 96.4% (5799) were male. Mean (±SD) age of men was 33.73 (±9.95) and for women was 32.54 (±12.36). For both men and women majority educational status was middle/secondary school (69.05% and 52.02; respectively). 5305 (99.4%) of the injured subjects survived and 33 (0.6%) had died.

### Univariate analyses

The effect of different independent factors on death of subjects understudy are also shown in Table 1. As Table 1 shows, the annual incidence of death was higher in injured subjects whose job tenure were higher than 1 year (0.9% vs 0.3%; *p* = 0.01). The annual incidence of death due to electrical shock was the highest (7.40%) in comparison with falling, collision, throw and hit (0.5%), Others (Trapping & Excessive force & Other reasons) (0.6%) (*p* < 0.001). Also, incidence of death in subjects with other reasons of trauma (0.87%) was higher in compare to the other parts of body trauma (*p* < 0.001).

**Table 1:** Frequency, percentage and univariate analyzing results for all independent variables by consequence, 2013

Factors	Consequence				Total	*P*-value
	Survived		Dead			
	*N*	%	*N*	%		
Sex						0.645
Male	5065	99.4	33	0.6	5065	
Female	219	100	0	0.0	219	
Education						0.551
Not graduate from high school	2248	99.4	15	0.6	2263	
Graduated from high school	2619	99.5	14	0.5	2633	
Age (years)						0.170
< 45	4321	99.5	23	0.5	4344	
> = 45	605	99.0	6	1.0	611	
Current Job Tenure (years)						0.01
<=1	1684	99.1	15	0.9	1699	
> 1	2198	99.7	6	0.3	2204	
Injury Cause						< 0.001
Electrical shock	63	92.6	5	7.4	68	
Falling & Collision & Throw & Hit	3053	99.5	16	0.5	3069	
Others^a^	1962	99.4	11	0.6	1973	
Referred location						0.481
Healthcare home	1629	99.3	12	0.7	1641	
Hospital	661	99.7	2	0.3	663	
Other referred location	193	99.5	1	0.5	194	
Occupation						0.351
Precision Production, Craft or Repair	482	99.6	2	0.4	484	
Service	353	98.6	5	1.4	358	
Farming, Forestry & Fishing	228	99.1	2	0.9	230	
Operators, Fabricators and Laborers	2529	99.5	13	0.5	2542	
Technicians and Related Support	41	100.0	0	0.0	41	
Professional Specialty	152	100.0	0	0.0	152	
Sales	6	100.0	0	0.0	6	
Exec, Administrative or Managerial	27	100.0	0	0.0	27	
Administrative Support or Clerical	217	99.5	1	0.5	218	
Shift work						0.292
Day shift	3237	99.5	17	0.5	3254	
Evening shift	1095	99.3	8	0.7	1103	
Night shift	481	99.0	5	1.0	486	
Season						0.056
Spring	1159	99.9	1	0.1	1160	
Summer	1285	99.4	8	0.6	1293	
Fall	1305	99.2	10	0.8	1315	
Winter	1421	99.4	9	0.6	1430	
Accident day						0.847
First 10 days	1723	99.5	8	0.5	1731	
Second 10 days	1742	99.3	13	0.7	1755	
Third 10 days	1695	99.6	7	0.4	1702	
Damaged parts of the body						< 0.001
Head & neck	838	99.3	6	0.7	844	
Lower extremity	2507	99.4	14	0.6	2521	
Thorax	258	99.5	1	0.5	259	
Abdomen & pelvis	153	99.4	1	0.6	154	
Upper extremity	1060	100.0	0	0.0	1060	
Other^b^	228	99.1	2	0.9	230	

^a^Trapping & Excessive force & Other reasons ^b^ Other kinds of body damage

### Exact multiple logistic regression analysis

It was observed that amongst injury causes, electric shock enforced the highest chance of death (OR = 7.04; 95% CI: 1.01–43.74; *p* = 0.02). However, chance of death for injured subjects with job tenure more than one year was nearly 80% lower (OR = 0.21; 95% CI: 0.05–0.70; *p* = 0.005) (Table 2).

**Table 2:** Exact multiple logistic regression analysis result

Factors	Odds ratio	95% confidence interval	P-value
Current Job Tenure (years)			
Less and equal to 1 year		Ref.	
More than 1 year	0.21	0.05–0.70	0.005
Injury Cause			
Others^a^		Ref.	
Electrical shock	7.04	1.01–43.74	0.02
Falling & Collision & Throw & Hit	0.52	0.10–1.85	0.27

^a^Trapping & Excessive force & Other reasons

### Years of life lost

In 2013 the life expectancy at birth for Iranian males was about 73 years, according to that cumulative YLL for 33 dead people were calculated 1289.12 years. Such that cumulative YLL in effect of falling was 389.11 years (*n*: 10; 95% CI: 33.24–44.57), collision & throw & hit, 279.60 years (*n*: 7; 95% CI: 27.07–52.79), trapping, 90.26 years (n: 2: 95% CI: 27.36–62.89), high or low temperature, 60.48 years (*n*: 2; 95% CI: -115.71, 176.20), electrical shock, 199.27 years (*n*: 5; 95% CI: 35.75–43.95), acute cause, 58.60 years (n: 2; 95% CI: -116.27, 174.83), others, 211.80 years (*n*: 5; 95% CI: 37.05–47.61).

**Fig. 1:**
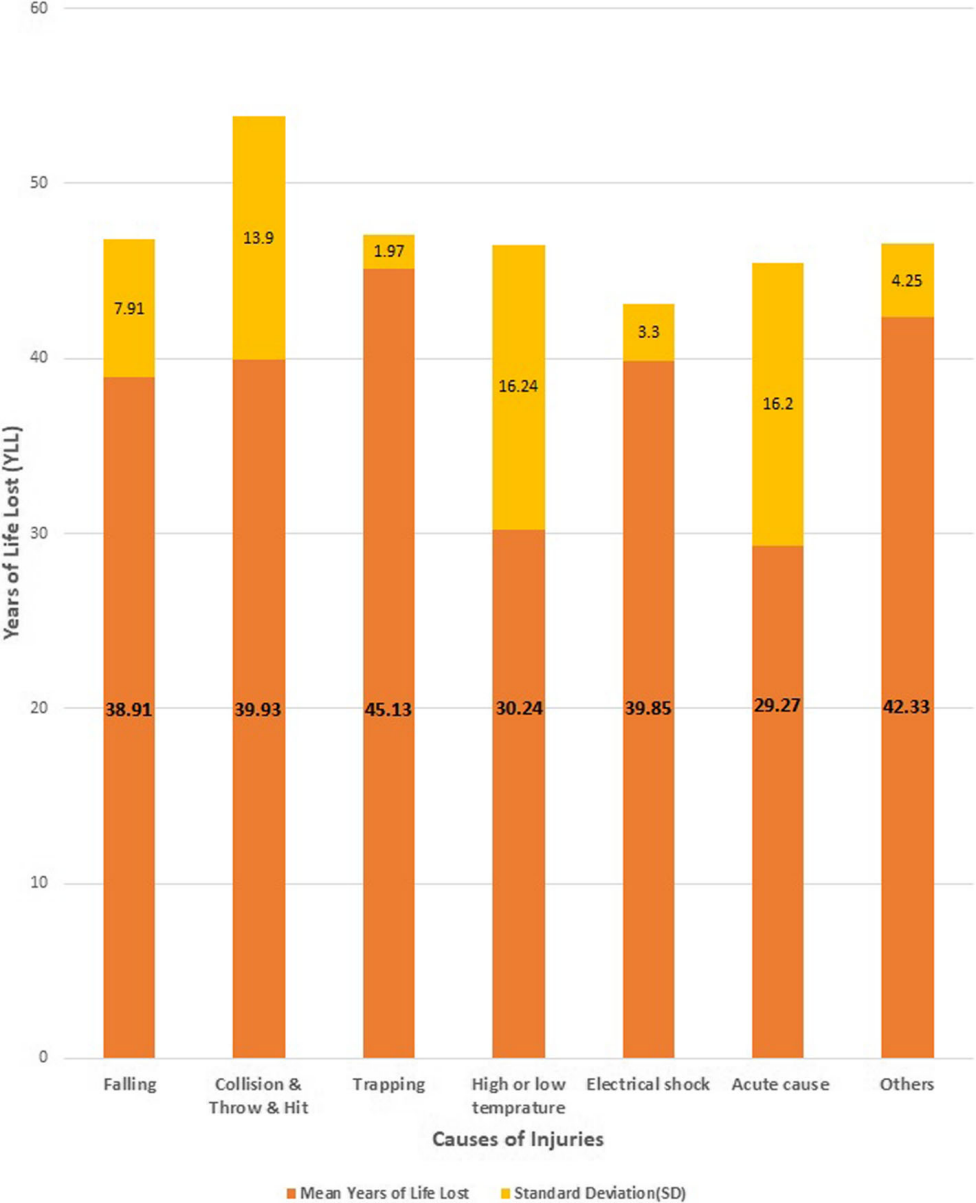
The mean (±SD) of YLL in effect of different causes of injuries

The mean (±SD) of YLL in effect of different COIs are shown in fig. 1. The difference between the means was not statistically significant (df: 2; F = 0.06; *p* = 0.08).

### Discussion

This study was the first study that conducted with using of a national wide data related to the occupational accidents in Iran. The main goal of this study was examination of relationships between the fatal occupational accidents and various socio-demographic factors, factors related to the work characteristics, and other available factors.

The multivariate results (Exact multiple logistic regression analysis) showed that current job tenure with fatal occupational accidents is associated. As expected, workers with less or equal to 1-year job tenure had more death (*N* = 15) in comparison to the workers with more than 1-year job tenure. It seems to work experience has protective effect on the happening of fatal accidents. Related to this, a study conducted by Chung J. and et al., positive correlation between age and work experience was observed (*p* < 0.01, correlation coefficient: 0.31) [15]. Also the study of J-M. Cellier and et al. indicated that low experience in beginning workers at comparison with intermediate workers, cause more frequent and seriousness of accidents [16]. The studies that were carried out in Korean, Chinese, Japanese, western cuisine restaurants showed that in all countries having longer period of work experience cause persons less to be injured by accidents [17]. Also, several studies have shown due to the effects of subcontracted work, labor turnover and short-term employment, new and inexperienced workers have the greatest risk of fatal occupational injury [18–20]. The question is, how work experience decreases the happening of accidents or seriousness of accidents, it may be related with the worker’s knowledge about job risks. For people who work in a company the knowledge about the risks which are specific to that place, increase with the passage of time so maybe this knowledge plays a protective role. On the other hand, Salthouse’s study revealed that the job experience can be effected basic cognitive processes or job performance positively [21].

As can see in Table 1 “falling & collision & throw & hit” is responsible for 16 deaths in total of 33 recorded deaths. But Exact multiple logistic regression analysis showed that just electrical shock as a cause of injury has statistical significant relationship with response variable. As shown in Table 1, electrical shock has more percentage of deaths (7.4%) in comparison to falling & collision & throw & tit group (0.5%) so this finding was not far from the mind. According to the Y.-H. Lin and et al. study, in dead male workers, falls (38.2%), electric shock (14.5%), collapse (11.3%) were the most leading causes of death [22]. In our study and in the dead workers like Y.-H. Lin and et al. study, falling & collision & throw & hit group was the first reasons of death so that it was responsible for 48.48% of deaths. Also our results were generally consistent with findings of Im and et al. study results. They found that in the construction industry in Korea, falling is the most frequent (52.7%) cause of fatal injuries. Also they found that deaths due to structural collapse and electric shock in construction industry in compare to the other industries is significantly higher [23]. In line with the findings of our study Cheng et al. reported that at small construction enterprises, the reason for 58 % of occupational accidents is falls and tumbles while electrical shock was responsible for 8% of occupational accidents [24]. It seems that lack of appropriate safeguards in result of inadequate safety management or not using of personal protection equipments are the probable reasons for the increase in the rates of falling, electrical shocks and so on.

In the calculation of the YLL because of any data was not registered for the dead women so we just did this calculation for men. Although because of cultural status and exist of some cultural limitations for woman in Iran this phenomenon was expectable. As we expected women in comparison with men employ mostly in less hazardous occupations in Iran so fatal occupational accidents less happen to them. Cohen et al. reported that average years of potential life lost (YPLL) per occupational fatality for workers employed in the remediation sites is 38.09 years [25]. In this study we found that the average YLL for each dead worker is about 39.06. A study conducted by mohammadfam et al. in Tehran (capital of Iran) showed that cumulative years of life lost for all dead insured workers because of occupational fatalities is 7552 years such that the average YLL for each dead worker was about 32.69 years [26]. It seems that difference in years studied and number of years studied are two main reasons to make differences in findings.

The primary interest of researchers was to investigate the effects of work organization factors like: work-family interference, management employee relations, organizational effectiveness, safety climate and job content on the fatal consequence of occupational accidents but unfortunately related factors were not recorded in the registry system of the Ministry of Health and Medical Education of Iran. Also, access to the one year of data related to the occupational accidents was other limitation of our study. So we suggest that in addition to the factors that investigated in this study other mentioned factors to be studied in the future research. Also investigation of fatal occupational accidents and related factors in the passing of time for studying of trends will be interested.

## Conclusions

Based on the findings, it seems that between independents variables, current job tenure (years) and injury cause affected fatal consequence of occupational accidents in Iran. The risk of death for workers have current job tenure more than 1 year in comparison to others is less. While the risk of death for electrical shock as a cause of injury in comparison to the other causes, is high. About YLL it was determined that between COIs, falling is the main cause of years of life lost in Iranian workers. So it seems that employing of workers who have more than one work experience in a specific job and using of appropriate safeguards against the falling and electrical shocks could reduce the frequency of fatal occupational accidents so in result the YLL will be reduced.

## Abbreviations

COIs: Causes of Injuries
ILO: International Labour Organization.
SD: Standard Deviation.
YLL: Years of Life Lost.
